# Effect of infections, DNA methylation and telomere length on frailty trajectories in hospitalized older patients: the INFRAGEN study protocol

**DOI:** 10.1186/s12877-025-06194-z

**Published:** 2025-07-23

**Authors:** Wenxiang Guo, Leonardo Bencivenga, Bruno Micael Zanforlini, Chiara Curreri, Maria Cristina Ferrara, Benedetta Maisano, Luca Tinelli, Laura Andreea Ceparano, Raffaella Merenda, Chiara Cosma, Lucia Manfron, Nicolò Gentili, Silvia Sturani, Monica Cardi, Alice Campion, Benedetta Berardi, Martina Lombardi, Elisabetta D’Aversa, Francesca Salvatori, Veronica Tisato, Joanne Vanessa Vargas, Grazia Daniela Femminella, Donato Gemmati, Giuseppe Sergi, Giuseppe Bellelli, Paolo Mazzola, Caterina Trevisan, Stefano Volpato, the INFRAGEN working group, Alessandra Cannata, Monica Cardi, Giulia Barrile, Marianna Boccafogli, Lisa Marzano, Dorotea D’Angelo, Francesca Remelli, Maria Cristina Ferrara, Beatrice Tonus, Giulia Cederle, Martina Marelli, Camilla Tocci, Giorgio Mauri, Leonardo Barbieri, Alberto Saporito, Flavia Sandi, Greta Tavecchi, Carola Arighi, Martina Sticchi, Gianluca Negro Cusa, Chiara Milan, Cristina Bianchi, Sofia De Marco, Barbara Torsello, Bruno Micael Zanforlini, Marina De Rui, Elena Maria Bicego, Vitalba Bivona, Nicola Rampon, Serena Lorenzo, Giorgia Longo, Marta Caenaro, Roberta Andreose, Simone Agostini, Claudia Cappellari

**Affiliations:** 1 School of Medicine and Surgery, University of Milano-Bicocca, Monza, Italy; 2 Department of Translational Medical Sciences, University of Naples “Federico II”, Naples, Italy; 3 Department of Medicine (DIMED), University of Padua, Via Giustiniani 2, 35128 Padua, Italy; 4 Geriatric Unit, Azienda Ospedale Università Padova, Via Giustiniani 2, 35128 Padua, Italy; 5 Department of Medical Science, University of Ferrara, Ferrara, Italy; 6 Geriatrics Unit, Azienda Ospedaliero-Universitaria of Ferrara, Ferrara, Italy; 7 Department of Translational Medicine, University of Ferrara, Ferrara, Italy; 8 Centre Haemostasis & Thrombosis, University of Ferrara, Ferrara, Italy; 9 Acute Geriatric Unit, Fondazione IRCCS San Gerardo Dei Tintori, Monza, Italy; 10 Aging Research Center, Karolinska Institutet, Stockholm, Sweden

**Keywords:** Frailty, Infections, Epigenetic factors, Genetics, Inflammation mediators, Longitudinal study

## Abstract

**Background:**

Infectious diseases are among the most common causes of hospitalization in older adults and may lead to a high burden on the individual’s health and healthcare system. However, it is unclear whether and to which extent these events might affect frailty, fastening its development or hampering its reversion. The aims of the INFRAGEN project are 1) to assess the impact of acute infections on frailty trajectories in older inpatients, and 2) to evaluate the modifying effect of sociodemographic, clinical, functional, and genetic/epigenetic factors on that association.

**Methods:**

INFRAGEN is a multicenter prospective observational study that will be conducted in the acute Geriatric Units of four Italian centers (Ferrara, Padova, Monza, and Napoli). The project will involve individuals aged ≥ 70 with no or mild-to-moderate pre-admission frailty (Clinical Frailty Scale [CFS] < 6) and diagnosis of acute infectious diseases at the time of hospital admission or during hospitalization. For each participant, we will record data concerning the multidimensional geriatric assessment and the type and severity of infectious diseases (diagnosed according to ICD-9 codes). Blood samples will be collected to assess Global DNA methylation, Leukocyte Telomere Length (LTL), and levels of circulating markers associated with biological processes related to frailty (inflammatory state, dysmetabolism, brain modifications, and oxidative stress). Frailty status will be evaluated through the CFS and Frailty Index at admission (referring to the 2 weeks before hospitalization), hospital discharge, and after 3 months. In a subsample, genetic/epigenetic analyses will also be performed at the 3-month follow-up.

**Discussion:**

INFRAGEN will contribute to exploring the complex pathophysiologic mechanisms of frailty in the context of infections in older adults through a translational approach.

**Trial registrations:**

NCT06430073 (ClinicalTrials.gov); Registration date: 2024–05-28.

## Background

Frailty, a geriatric syndrome characterized by reduced biological reserves and increased vulnerability to stressors, represents a cornerstone of geriatric clinical practice and research. As the global population ages, this syndrome is expected to constitute an increasingly pressing public health problem. It is associated with several adverse outcomes, including acute and chronic conditions, disability, cognitive decline, and mortality [1, 2].

Frailty exacerbates age-related changes in the immune system and chronic inflammation [3], presumably increasing the individual vulnerability to infections. In turn, infections can trigger inflammatory responses and aggravate the detrimental effects of hospitalization on the individual’s resilience and functional status [4]. Previous studies have shown a link between frailty at hospital admission and adverse outcomes in patients with infectious diseases, including an increased risk of hospital readmission and mortality [5] and poorer medium-term functional trajectories [6]. However, the intricate relationship between frailty and acute infections remains poorly understood, as does the impact of acute infections on this geriatric syndrome.

To date, research focuses mainly on COVID-19 and shows that infection significantly worsens frailty, particularly in hospitalized patients [7] and those with pre-existing frailty [8]. Moreover, studies investigating changes in frailty during hospitalization have generally assessed frailty only at admission, neglecting the pre-admission status and often overlooking the potential impact of infectious diseases [9, 10].

An interesting aspect to consider in the context of the interplay between frailty and infections is the role of genetic and epigenetic biomarkers. Emerging research points to the potential involvement of factors, such as global DNA methylation and telomere length, in the development and progression of frailty [11–13]. However, further studies are necessary to gain a deeper understanding of their roles and potential clinical implications.

The INFRAGEN prospective multicenter study aims to evaluate the impact of infections on changes to frailty in hospitalized older patients, focusing on clinical, biochemical, genetic, and epigenetic markers at hospital admission. Moreover, the study will examine whether changes in frailty over three months are linked to epigenomic DNA modifications in a subgroup of patients.

The specific aims of the study will be: a) to assess the impact of infections—both with and without systemic inflammatory response—on frailty profiles from pre-admission to hospital discharge in older patients with no to mild pre-admission frailty (*primary aim*); b) to examine the link between changes in frailty and genetic/epigenetic markers, such as global DNA methylation and telomere length, exploring whether these biomarkers can predict the course of in-hospital and post-discharge frailty; c) to evaluate the trend of circulating inflammatory and aging-related biomarkers in modulating the impact of infections on frailty progression; and d) to assess the impact of infections on frailty trajectories over a three-month follow-up after discharge, and the modifying role of sociodemographic, clinical and (epi)genetic factors.

While previous research has linked hospitalizations to a decline in physical and cognitive health, leading to frailty in older patients, limited evidence exists on how infections specifically drive these changes. We hypothesize that patients experiencing a higher number and severity of acute infections, especially those with underlying systemic inflammation, may show a steeper functional decline during and after hospitalization. Additionally, we expect that genetic, epigenetic, and clinical factors—such as older age, multimorbidity, polypharmacy, specific DNA methylation and telomere length patterns—may modify the progression of frailty in response to infections.

## Methods/design

### Study design and participants

The INFRAGEN study is a prospective, observational multicenter study involving the Acute Geriatric Units of four Italian centers: S. Anna University Hospital in Ferrara (Emilia-Romagna region, Italy), Padua University Hospital (Veneto region, Italy), IRCCS Foundation San Gerardo dei Tintori in Monza (Lombardy region, Italy), and Federico II University Hospital (Campania region, Italy).

Participants will be selected based on the following criteria: age ≥ 70 years, non-frailty or mild frailty status before admission (CFS < 6), and confirmed diagnosis of acute infectious diseases at the time of hospital admission or during the hospital stay, according to specific ICD-9 codes, with or without a systemic inflammatory response.

Terminally ill patients with an estimated life expectancy of less than 3 months, those with moderate to severe frailty, and individuals who do not wish to participate in the study or complete follow-up assessments will be excluded.

Written informed consent will be obtained from all participants.

### Sample size calculations

Based on previous reports on the topic [10], we estimated that a total sample of at least 340 older adults with acute infections would be sufficient to detect a minimal clinically significant difference in Frailty Index (FI) of at least 0.03 points from the pre-admission status to hospital discharge, with an alpha of 0.05, and a power of 80%.

### Study procedures

**Fig. 1:**
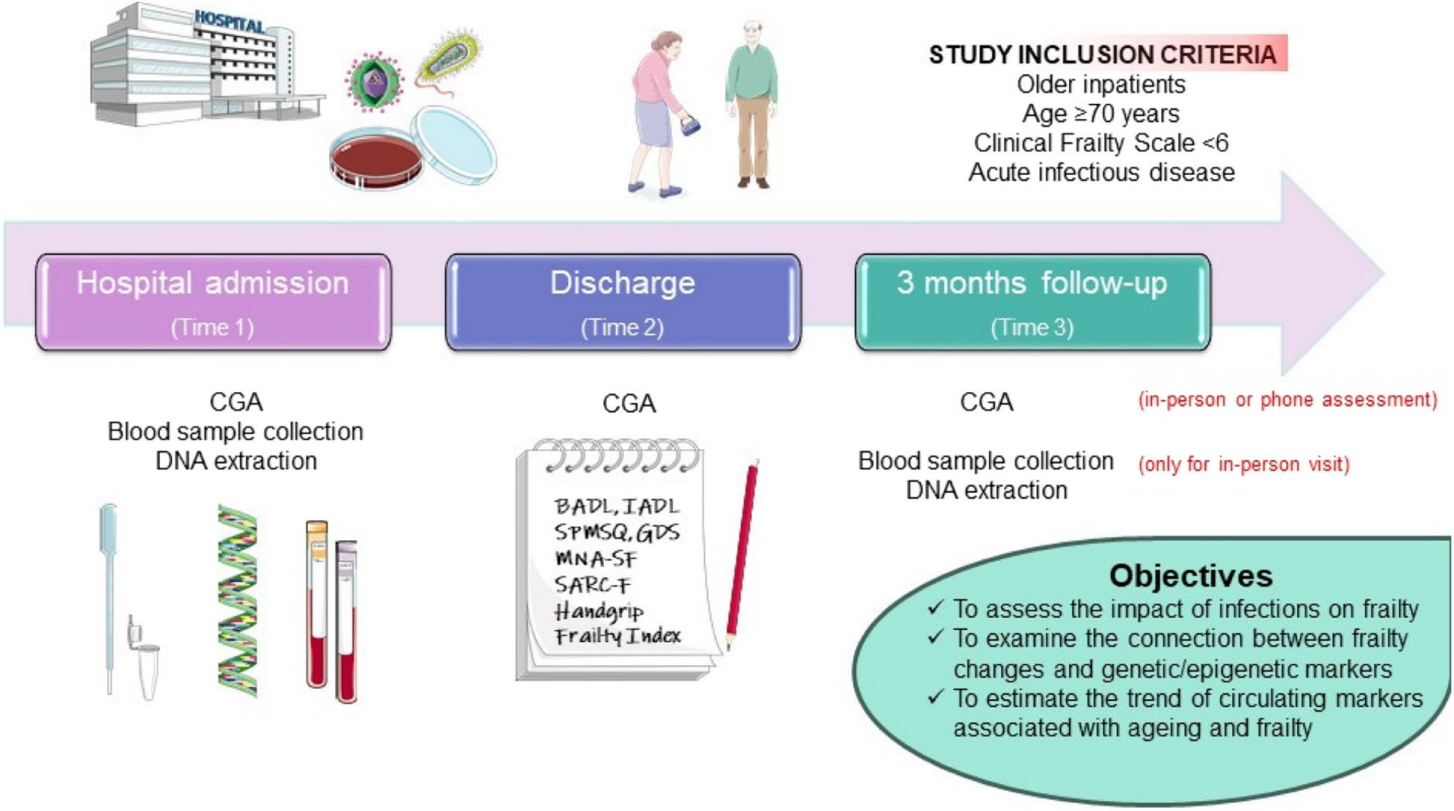
Graphical summary of the INFRAGEN study protocol

Data will be collected using the RedCap platform. The study will include three assessment points (Fig. 1): hospital admission (Time 1), discharge (Time 2), and three months after hospital discharge (Time 3).

Time 3 assessment will be performed through an outpatient follow-up visit, home visit, or telephone interview, depending on the centers’ resources and the patient’s characteristics and availability.

Each assessment will include face-to-face interviews, administration of validated scales and questionnaires, physical tests, and blood sampling (the latter only at Time 1 and 3, and limited to the subgroup undergoing outpatient clinic visits).

The data collected from each participant are briefly described below.

#### Sociodemographic factors

Sociodemographic information includes age, sex, pre-admission setting (home, family home, long-term care, other hospital wards), and assistance needs from home.

#### Comprehensive geriatric assessment

Functional status will be assessed using the validated Basic Activities of Daily Living (BADL) and Instrumental Activities of Daily Living (IADL) scales [14, 15]. The presence of cognitive deficits will be evaluated using the Short Portable Mental Status Questionnaire (SPMSQ) [16].

Chronic medical illness burden will be quantified using the Cumulative Illness Rating Scale (CIRS) [17]. Delirium screening will be performed using the 4AT screening tool [18].

Nutritional status and sarcopenia screening will be evaluated using the Mini Nutritional Assessment Short Form (MNA-SF) and SARC-F [19, 20]. Anthropometric measurements, including body weight, height, and calf and arm circumferences, will be recorded for each participant. Pressure sore risk will be determined using the Exton-Smith Scale (ESS) [21]. Depressive symptoms will be assessed using the Geriatric Depression Scale (GDS) [22], and physical performance through handgrip strength and 4-m walking speed, only for those who are able to walk independently or with walking aids.

Furthermore, we will collect data on the patient’s medication history, including medications used chronically before admission and those prescribed at discharge. Information on vaccination history, alcohol consumption, and smoking status will also be obtained.

#### Infectious disease

The presence of infectious diseases will be determined through routine clinical, radiological, biochemical, and microbiological analyses and coded according to the following ICD-9 codes: 001–139 (Infectious diseases); 460–466 (Acute respiratory infections); 480–487 (Pneumonia, flu disease); 320–326 (Nervous system infections); 540–543 (Appendicitis); 590, 595.0, 595.4, 599.0, 597 (Urinary system infections); 601, 604 (Prostatitis, orchitis, and epididymitis); 616, 574.0, 571.1 (Gynecological system infections); 572.0, 573.1, 573.2 (Acute hepatitis, liver abscess); 575–576.1 (Cholecystitis, cholangitis).

Each documented infectious disease will be characterized by its etiology (if known), and the prescribed antimicrobial therapy will be recorded, including the specific drug and duration. Any concomitant or subsequent infectious diseases arising during hospitalization will be similarly documented. In addition, the Sequential Organ Failure Assessment (SOFA) score will be calculated for each patient to assess infections’ severity [23].

#### Frailty status

Frailty will be evaluated using the Primary Care – Frailty Index (PC-FI) [24] and the Clinical Frailty Scale (CFS) [25]. The PC-FI is a reliable and validated tool developed in Italian older adults to assess frailty in the primary care setting. It was chosen considering that both the pre-admission and post-discharge assessment will involve older adults in primary care. In particular, pre-admission frailty status will be derived retrospectively to reflect the status of participants two weeks before hospital admission.

Frailty assessment will be repeated 48 h before hospital discharge and at a 3-month follow-up.

#### Genetic and epigenetic analyses

At Time 1 and Time 3 (only for a subsample of participants), blood samples will be collected and processed for plasma and blood cell stocking for DNA extraction.

DNA extraction from whole blood will be performed by Automated Genomic DNA Purification EZ1 XL machine (QIAGEN).

Global DNA methylation assessment will be performed by “highly quantitative pyrosequencing” technique as genome-wide DNA methylation levels and as gene promoter associated CpG islands utilizing selected age-related methylation marker loci and at LINE-1, Alu, Sat-alpha repetitive elements (as a surrogate for global genome methylation) as previously described [26–28]. Results will be also confirmed by internal Illumina Infinium 450 k beadchip array.

Leukocyte Telomere Length (LTL) will be determined using quantitative real-time PCR (QuantStudio™ 3 System; Applied Biosystems) according to the method of Cawthon [29], the 36B4 will be used as a single copy gene reference (SCR), and the ratio Telomere/36B4 (T/S) will be estimated as indicator of LTL [2 − ∆Ct (△Ct = Ct TL − Ct 36B4)].

#### Biochemical analyses

We will monitor the plasma levels of circulating markers associated with key biological processes related to frailty, such as (1) pro-inflammatory state (including but not limited to TNF-a, IL6, CRP, TRAIL/OPG, CXCL10, MCP-1); (2) metabolic/endocrine imbalance (including but not limited to Cystatin-C, IGF-1); (3) functional brain modifications (including but not limited to BDNF, CSF); (4) oxidative stress (including but not limited to PON-1, Homocysteine).

Analysis will be performed using Luminex Technology, a platform allowing the simultaneous detection and quantification of multiple secreted proteins, including cytokines, chemokines, and growth factors. Customized panels will be generated with targets of interest.

Ox-stress markers will be analyzed using fluorimetric/ELISA assays.

Selected inflammatory markers will be monitored by ProQuantum High-Sensitivity immunoassays (TermoFisher, Waltham, MA USA) to achieve a highly sensitive assay to detect lower protein levels.

### Statistical analysis

The baseline characteristics of the study participants will be described using count and proportion for the categorical variables and mean and standard deviation, or median and interquartile ranges, for the quantitative variables, according to their distribution. These characteristics will be compared between participants by type of infection and the presence of systemic inflammatory response through the ANOVA, Student t-test, Kruskal Wallis test, or Chi-squared test, as appropriate.

The intra-individual change in FI between the pre-admission status and the patient’s condition at hospital discharge will be evaluated through the Student t-test for paired samples and then with linear mixed models. The latter analysis will be set with a random intercept and slope and adjusted for potential confounders in the association between infectious diseases and frailty changes, e.g., sociodemographic information, study center, comorbidities, length of hospital stay, and in-hospital clinical events (unrelated to the infection). The mean adjusted change in FI between pre-admission status and hospital discharge will be expressed as ß coefficient and 95% confidence interval (95% CI). Moreover, we will test whether the presence of systemic inflammation will modify the average intra-hospital change in FI. As secondary analyses, we will investigate which infection-related characteristics will be associated with higher in-hospital mortality (through the Kaplan-Mayer method and Cox regression analysis), longer hospital stay (with linear regressions), and discharge setting (with multinomial logistic regression).

The impact of infectious diseases (distinguished by number, site and severity) on frailty changes will be analyzed with linear mixed models, considering the beta coefficients (95% CI) for the interaction infection*time. Additional analyses will be performed to test whether sociodemographic, functional, clinical, and genetic/epigenetic factors interact with infectious diseases to influence the course of frailty over time.

The patterns of global DNA methylation will be assessed in duplicate for each sample and expressed in percentage as the mean obtained by the two evaluations and considered valuable with a discrepancy < 2%. Methylation percentages will be stratified into quartiles, and the combined middle two quartiles will be used as the reference category to identify potential correlations at the top or bottom of the distribution with the clinical phenotypes. As a measure of the relative Telomere length, the ratio of the telomere repeat copy number to the number of single-copy genes (T/S ratio) will be determined by quantitative PCR using the single-copy gene 36B4 for reference and a standard curve. Quality controls and assay validation tests will be assessed by official commercially recognized standards (Qiagen, LifeTechnology). The association of global DNA methylation pattern and telomere length with the frailty trajectories will be evaluated through a linear mixed model, adjusted for potential confounders. Appropriate subgroup and interaction analyses will be performed to explore the possible modifying effects by sex, the presence of the most prevalent clinical conditions, and infection-related factors (i.e. number, site, and severity of infections) on the studied associations.

## Discussion

This project will investigate the relationship between infections in hospitalized older patients and frailty trajectories over time. Frailty is a cornerstone of geriatric clinical practice because it is associated with adverse health outcomes, including hospitalization, length of hospital stay, falls, delirium, and mortality [2, 30]. The INFRAGEN project will provide novel contributions to this area of research in four main ways.

First, the study will quantify the impact of infections on the change in individuals’ frailty status from pre-admission to hospital discharge. Evidence from the SARS-CoV-2 pandemic suggests that infectious diseases can substantially promote frailty development even beyond 3 months of acute illness [7, 8]. However, data on how the most common types of infection, their severity, the occurrence of multiple infectious diseases, and their related level of systemic inflammation may influence the progression of frailty in older adults are lacking. Furthermore, there is some heterogeneity in the definitions of frailty adopted across various studies. Second, the project will explore the interaction between infections and in-hospital frailty status in influencing adverse endpoints, such as in-hospital mortality, length of hospital stay, and discharge destination. For instance, sepsis in frail patients has been shown to worsen mortality compared to non-frail patients [31], and other infections such as pneumonia, COVID-19, or influenza may have similar detrimental impacts [32–34]. Additionally, frailty is linked to a higher risk of urinary tract infections [35]. Third, the project will assess whether global DNA methylation and telomere length patterns at hospital admission are predictors of in-hospital and post-discharge frailty trajectories. In the literature, these epigenomic and genomic markers have been linked to cellular senescence and aging [36]. For example, shorter LTL have been associated with increased risk of infections, various malignant tumors (esophageal cancer, lymphoid and myeloid leukemia), and mortality from cardiovascular, respiratory, digestive, and musculoskeletal diseases and COVID-19 [11]. However, the role of these genomic markers in predicting frailty or functional changes has not been fully investigated [13]. This project will allow us to explore whether DNA methylation and telomere length variations are involved in the pathogenetic processes leading to frailty, especially following an infection. Notably, epigenetic modifications and telomere attrition (dynamic epigenomics) are sensitive to exogenous and endogenous stressors, which strongly relates to an individual’s baseline epigenome and their personal response to stressors, thus accounting for the relationship between epigenomic patterning and developmental trajectories or disease [37]. Although a 3-month period may be a short time interval to detect changes in LTL, we cannot rule a negative impact of infectious diseases and inflammatory processes on this aspect in vulnerable individuals, such as older inpatients. Moreover, blood cells composition of the included participants is expected to change between in-hospital and post-discharge assessments, and this issue may further influence LTL.

Finally, the INFRAGEN project will clarify which inflammatory markers (e.g., Interleukins 1 to 18, GDF15, IF1, TNFR, MCP-1) are mostly predictive of frailty progression and adverse health-related outcomes in older adults with acute infections. Frail individuals are known to have significantly reduced innate immune function, T-cell activity, antibody production, and a persistent state of chronic mild inflammation. However, longitudinal studies have not demonstrated a clear association between higher inflammatory levels and the onset of frailty [4]. Research in this field may help contribute to the current literature on the relationship between inflammatory markers, frailty pathophysiological pathways, and patient clinical phenotypes. By enrolling patients with no or only mild frailty before hospital admission and who are affected by acute infections generally triggering inflammation, we will be able to derive a risk stratification of frailty development based on inflammatory, genetic/epigenetic and clinical factors.

The INFRAGEN project has several strengths, including multicenter patient enrollment, standardization of biochemical and genomic analyses in a central laboratory, and broad multidomain characterization of older patients. Moreover, combining clinical and pre-clinical research expertise will allow us to provide novel findings that will contribute to the pathophysiological understanding of frailty and may have concrete implications for clinical practice. Potential critical points may include the recruitment of patients with no or mild frailty in acute geriatric units, which may be challenging because older adults needing hospitalization are often clinically complex and frail at the time of admission. However, the coordination of participating centers may transform these potential challenges into opportunities to investigate understudied topics. Another limitation of our study is the relative homogeneity of the older Italian population, which is still predominantly Caucasic and may make our results not generalizable to other ethnic groups. The generalizability of the project findings may also be affected by the involvement of only Italian Geriatric units. However, these wards exhibit some differences in terms of hospital organization, infection epidemiology, and regional healthcare practices, which partly account for the heterogeneity of healthcare systems.

Regarding dissemination activities, the project results will be published in scientific papers submitted to international biomedical journals. Additionally, study team members will participate in national and international conferences to further disseminate the project findings and their potential translational clinical implications. These efforts will highlight the importance of stimulating clinical and pre-clinical research in this field.

## Conclusions

The INFRAGEN study will contribute to understanding the role of infections and inflammation in prompting the development of frailty in older adults. Additionally, it will explore whether genetic and epigenetic factors, such as global DNA methylation and telomere attrition, may modify the impact of infections on frailty trajectories during hospitalization and after hospital discharge. Therefore, the results of this project will help clarify the underlying pathophysiological mechanisms of frailty and stratify the risk of frailty and other adverse outcomes (e.g. mortality, prolonged hospital stay, repeated hospital readmissions, infection recurrence, or reduced independence) in older inpatients with acute infections.

## Data Availability

No datasets were generated or analysed during the current study.

## Abbreviations

CFS: Clinical Frailty Scale
FI: Frailty Index
ICD-9: International Classification of Diseases, Ninth Revision
BADL: Basic Activities of Daily Living
IADL: Instrumental Activities of Daily Living
SPMSQ: Short Portable Mental Status Questionnaire
CIRS: Cumulative Illness Rating Scale
MNA-SF: Mini Nutritional Assessment Short Form
ESS: Exton-Smith Scale
GDS: Geriatric Depression Scale
SOFA: Sequential Organ Failure Assessment
LTL: Leukocyte Telomere Length

## References

[1] Adja KYC, Lenzi J, Sezgin D, O’Caoimh R, Morini M, Damiani G, et al. The Importance of Taking a Patient-Centered, Community-Based Approach to Preventing and Managing Frailty: A Public Health Perspective. Front Public Health. 2020;8: 599170. 33282818 10.3389/fpubh.2020.599170 PMC7689262

[2] Chen X, Mao G, Leng SX. Frailty syndrome: an overview. Clin Interv Aging. 2014;9:433–41. 24672230 10.2147/CIA.S45300 PMC3964027

[3] Iwai-Saito K, Shobugawa Y, Aida J, Kondo K. Frailty is associated with susceptibility and severity of pneumonia in older adults (A JAGES multilevel cross-sectional study). Sci Rep. 2021;11:7966. 33846416 10.1038/s41598-021-86854-3 PMC8041848

[4] Soysal P, Stubbs B, Lucato P, Luchini C, Solmi M, Peluso R, et al. Inflammation and frailty in the elderly: a systematic review and meta-analysis. Ageing Res Rev. 2016;31:1–8. 27592340 10.1016/j.arr.2016.08.006

[5] Kundi H, Wadhera RK, Strom JB, Valsdottir LR, Shen C, Kazi DS, et al. Association of frailty with 30-day outcomes for acute myocardial infarction, heart failure, and pneumonia among elderly adults. JAMA Cardiol. 2019;4:1084–91. 31553402 10.1001/jamacardio.2019.3511 PMC6763977

[6] Park CM, Dhawan R, Lie JJ, Sison SM, Kim W, Lee ES, et al. Functional status recovery trajectories in hospitalised older adults with pneumonia. BMJ Open Respir Res. 2022;9: e001233. 35545298 10.1136/bmjresp-2022-001233 PMC9096550

[7] Müller I, Mancinetti M, Renner A, Bridevaux P-O, Brutsche MH, Clarenbach C, et al. Frailty assessment for COVID-19 follow-up: a prospective cohort study. BMJ Open Respir Res. 2022;9: e001227. 35459694 10.1136/bmjresp-2022-001227 PMC9035838

[8] Greco GI, Noale M, Trevisan C, Zatti G, DallaPozza M, Lazzarin M, et al. Increase in Frailty in Nursing Home Survivors of Coronavirus Disease 2019: Comparison With Noninfected Residents. J Am Med Dir Assoc. 2021;22:943-947.e3. 33757725 10.1016/j.jamda.2021.02.019 PMC7898983

[9] Jäger J, Sieber CC, Gaßmann K-G, Ritt M. Changes of a frailty index based on common blood and urine tests during a hospital stay on geriatric wards predict 6-month and 1-year mortality in older people. Clin Interv Aging. 2019;14:473–84. 30880928 10.2147/CIA.S191117 PMC6394369

[10] Basile G, Catalano A, Mandraffino G, Maltese G, Alibrandi A, Ciancio G, et al. Frailty modifications and prognostic impact in older patients admitted in acute care. Aging Clin Exp Res. 2019;31:151–5. 29946755 10.1007/s40520-018-0989-7

[11] Schneider CV, Schneider KM, Teumer A, Rudolph KL, Hartmann D, Rader DJ, et al. Association of telomere length with risk of disease and mortality. JAMA Intern Med. 2022;182:291–300. 35040871 10.1001/jamainternmed.2021.7804 PMC8767489

[12] Seligman BJ, Berry SD, Lipsitz LA, Travison TG, Kiel DP. Epigenetic age acceleration and change in frailty in MOBILIZE Boston. J Gerontol A Biol Sci Med Sci. 2022;77:1760–5. 35037036 10.1093/gerona/glac019 PMC9434439

[13] Vetter VM, Kalies CH, Sommerer Y, Spira D, Drewelies J, Regitz-Zagrosek V, et al. Relationship between 5 epigenetic clocks, telomere length, and functional capacity assessed in older adults: cross-sectional and longitudinal analyses. J Gerontol A Biol Sci Med Sci. 2022;77:1724–33. 35032170 10.1093/gerona/glab381

[14] Katz S, Ford AB, Moskowitz RW, Jackson BA, Jaffe MW. Studies of illness in the aged: the index of ADL: a standardized measure of biological and psychosocial function. JAMA. 1963;185:914–9. 14044222 10.1001/jama.1963.03060120024016

[15] Lawton MP, Brody EM. Assessment of older people: self-maintaining and instrumental activities of daily living. Gerontologist. 1969;9:179–86. 5349366

[16] Pfeiffer E. A short portable mental status questionnaire for the assessment of organic brain deficit in elderly patients. J Am Geriatr Soc. 1975;23:433–41. 1159263 10.1111/j.1532-5415.1975.tb00927.x

[17] Linn BS, Linn MW, Gurel L. Cumulative illness rating scale. J Am Geriatr Soc. 1968;16:622–6. 5646906 10.1111/j.1532-5415.1968.tb02103.x

[18] Bellelli G, Morandi A, Davis DHJ, Mazzola P, Turco R, Gentile S, et al. Validation of the 4AT, a new instrument for rapid delirium screening: a study in 234 hospitalised older people. Age Ageing. 2014;43:496–502. 24590568 10.1093/ageing/afu021 PMC4066613

[19] Kaiser MJ, Bauer JM, Ramsch C, Uter W, Guigoz Y, Cederholm T, et al. Validation of the mini nutritional assessment short-form (MNA-SF): a practical tool for identification of nutritional status. J Nutr Health Aging. 2009;13:782–8.19812868 10.1007/s12603-009-0214-7

[20] Malmstrom TK, Morley JE. SARC-F: a simple questionnaire to rapidly diagnose sarcopenia. J Am Med Dir Assoc. 2013;14:531–2. 23810110 10.1016/j.jamda.2013.05.018

[21] Bliss MR, McLaren R, Exton-Smith AN. Mattresses for preventing pressure sores in geriatric patients. Mon Bull Minist Health Public Health Lab Serv. 1966;25:238–68. 6013600

[22] Mitchell AJ, Bird V, Rizzo M, Meader N. Diagnostic validity and added value of the geriatric depression scale for depression in primary care: a meta-analysis of GDS30 and GDS15. J Affect Disord. 2010;125:10–7. 19800132 10.1016/j.jad.2009.08.019

[23] Vincent JL, Moreno R, Takala J, Willatts S, De Mendonça A, Bruining H, et al. The SOFA (Sepsis-related Organ Failure Assessment) score to describe organ dysfunction/failure. On behalf of the Working Group on Sepsis-Related Problems of the European Society of Intensive Care Medicine. Intensive Care Med. 1996;22:707–10. 10.1007/BF01709751 8844239

[24] Vetrano DL, Zucchelli A, Onder G, Fratiglioni L, Calderón-Larrañaga A, Marengoni A, et al. Frailty detection among primary care older patients through the Primary Care Frailty Index (PC-FI). Sci Rep. 2023;13:3543. 36864098 10.1038/s41598-023-30350-3 PMC9981758

[25] Rockwood K, Song X, MacKnight C, Bergman H, Hogan DB, McDowell I, et al. A global clinical measure of fitness and frailty in elderly people. CMAJ. 2005;173:489–95. 16129869 10.1503/cmaj.050051 PMC1188185

[26] Bollati V, Galimberti D, Pergoli L, Dalla Valle E, Barretta F, Cortini F, et al. DNA methylation in repetitive elements and Alzheimer disease. Brain Behav Immun. 2011;25:1078–83. 21296655 10.1016/j.bbi.2011.01.017 PMC3742099

[27] Tisato V, Silva JA, Scarpellini F, Capucci R, Marci R, Gallo I, et al. Epigenetic role of LINE-1 methylation and key genes in pregnancy maintenance. Sci Rep. 2024;14:3275. 38332006 10.1038/s41598-024-53737-2 PMC10853191

[28] Tisato V, Castiglione A, Ciorba A, Aimoni C, Silva JA, Gallo I, et al. LINE-1 global DNA methylation, iron homeostasis genes, sex and age in sudden sensorineural hearing loss (SSNHL). Hum Genomics. 2023;17:112. 38098073 10.1186/s40246-023-00562-9 PMC10722762

[29] Cawthon RM. Telomere measurement by quantitative PCR. Nucleic Acids Res. 2002;30: e47. 12000852 10.1093/nar/30.10.e47 PMC115301

[30] Hubbard RE, Peel NM, Samanta M, Gray LC, Mitnitski A, Rockwood K. Frailty status at admission to hospital predicts multiple adverse outcomes. Age Ageing. 2017;46:801–6. 28531254 10.1093/ageing/afx081

[31] Barbash IJ. Moving forward: frailty and adverse sepsis outcomes. Crit Care Med. 2022;50:880–2. 35485588 10.1097/CCM.0000000000005377

[32] Zhao L, Chen J, Zhu R. The relationship between frailty and community-acquired pneumonia in older patients. Aging Clin Exp Res. 2023;35:349–55. 36447006 10.1007/s40520-022-02301-x

[33] Remelli F, Volpato S, Trevisan C. Clinical features of SARS-CoV-2 infection in older adults. Clin Geriatr Med. 2022;38:483–500. 35868668 10.1016/j.cger.2022.03.001 PMC8934709

[34] Lees C, Godin J, McElhaney JE, McNeil SA, Loeb M, Hatchette TF, et al. Frailty hinders recovery from influenza and acute respiratory illness in older adults. J Infect Dis. 2020;222:428–37. 32147711 10.1093/infdis/jiaa092 PMC7336554

[35] Tang M, Quanstrom K, Jin C, Suskind AM. Recurrent urinary tract infections are associated with frailty in older adults. Urology. 2019;123:24–7. 30296501 10.1016/j.urology.2018.09.025 PMC8528015

[36] Chen BH, Carty CL, Kimura M, Kark JD, Chen W, Li S, et al. Leukocyte telomere length, T cell composition and DNA methylation age. Aging (Albany NY). 2017;9:1983–95. 28930701 10.18632/aging.101293 PMC5636670

[37] Ray M, Wallace MK, Grayson SC, Cummings MH, Davis JA, Scott J, et al. Epigenomic links between social determinants of health and symptoms: a scoping review. Biol Res Nurs. 2023;25:404–16. 36537264 10.1177/10998004221147300 PMC10404910

